# A Framework to Evaluate Wildlife Feeding in Research, Wildlife Management, Tourism and Recreation 

**DOI:** 10.3390/ani3040978

**Published:** 2013-10-11

**Authors:** Sara Dubois, David Fraser

**Affiliations:** Animal Welfare Program, University of British Columbia, 2357 Main Mall, Vancouver, BC, V6T 1Z4, Canada; E-Mail: david.fraser@ubc.ca

**Keywords:** animal welfare, conservation, framework, harm, intentional feeding, provisioning, public safety, wildlife

## Abstract

**Simple Summary:**

Human feeding of wildlife is a world-wide phenomenon with very diverse effects on conservation, animal welfare and public safety. From a review of the motivations, types and consequences of wildlife feeding, an evaluative framework is presented to assist policy-makers, educators and managers to make ethical- and biologically-based decisions about the appropriateness of feeding wildlife in the context of research, wildlife management, tourism and recreation.

**Abstract:**

Feeding of wildlife occurs in the context of research, wildlife management, tourism and in opportunistic ways. A review of examples shows that although feeding is often motivated by good intentions, it can lead to problems of public safety and conservation and be detrimental to the welfare of the animals. Examples from British Columbia illustrate the problems (nuisance animal activity, public safety risk) and consequences (culling, translocation) that often arise from uncontrolled feeding. Three features of wildlife feeding can be distinguished: the feasibility of control, the effects on conservation and the effects on animal welfare. An evaluative framework incorporating these three features was applied to examples of feeding from the literature. The cases of feeding for research and management purposes were generally found to be acceptable, while cases of feeding for tourism or opportunistic feeding were generally unacceptable. The framework should allow managers and policy-makers to distinguish acceptable from unacceptable forms of wildlife feeding as a basis for policy, public education and enforcement. Many harmful forms of wildlife feeding seem unlikely to change until they come to be seen as socially unacceptable.

## 1. Introduction

Feeding wildlife is a long-standing issue for wildlife managers and governments trying to reduce human-wildlife conflict [1,2,3]. Both unintentional and intentional feeding can cause harm to diverse wildlife species. Unintentional feeding occurs when wild animals are attracted to garbage, compost, landfills, gardens, fruit trees, pet food and other anthropogenic foods. Although these foods may improve welfare by reducing foraging needs in the short-term [4], in the long-term, anthropogenic foods can cause suffering [5,6], increased conflict with humans and the death of food-conditioned wildlife [2,7,8,9]. 

Intentional feeding of wildlife is necessary in captive environments where wild animals depend completely upon human husbandry (such as in wildlife rehabilitation). However, it also occurs across a spectrum of semi-captive and wild environments [10], the most widespread and socially accepted example being backyard bird feeding. Studies in Australia have investigated this popular activity to assess the extent of feeding, who feeds and the underlying motivations [11,12,13,14,15]; yet, the full ecological impacts of bird feeding are still poorly understood [16], and there is a lack of other comprehensive studies outside of Australia [17]. Globally, there are many other intentional feeding activities documented for species, like bears, ungulates, primates, sharks, dolphins and waterfowl; yet, few measure the long-term effects on the animals. 

A general perception is that feeding wildlife recreationally does not conflict with conservation goals and, in some situations, may appear to contribute toward them; however, little research has focused on assessing these beliefs. The science of animal welfare offers another approach by assessing whether feeding advances the quality of life of the individuals involved. Although animal welfare science has traditionally focused on harm to animals under direct human care (farm, companion and captive animals), the science is also applicable to unintentional and indirect harm to free-living wildlife [18,19]. Animal welfare assessment considers if an activity promotes physical and psychological well-being, prevents suffering and allows animals to live in ways suited to their natural adaptations [20]. Further, gauging the harm of feeding wildlife should also consider the severity of the welfare effects (*i.e.*, the number of animals affected, duration and the capacity of the animal to suffer) [21], which will vary according to the type of feeding and species involved. 

Incorporating animal welfare concerns alongside conservation goals may seem inappropriate, because conservation operates at the level of species and ecosystems, whereas animal welfare focuses on animals as individuals [22]. Yet, animal welfare and conservation share a common goal of reducing harm to animals and the common problems of increasing human population and industrialization, which threaten ecosystems, populations and individual animals [19]. Where human-wildlife conflicts emerge and direct conservation goals are not affected, framing wildlife management issues with an animal welfare perspective may assist in resolving issues, e.g., [23]. This paper reviews the literature on the motivations and types of intentional feeding, examines specific cases involving bears and deer and proposes an evaluative framework for assessing when feeding is justifiable.

## 2. Types of Wildlife Feeding: Motivations and Outcomes

### 2.1. Motivations for Feeding Wildlife

Motivations for feeding wildlife include benefits to animals and benefits to people. Suggested benefits to animals include improved survival and breeding rates [24,25] and increased public awareness, leading to support for conservation [26]. Reported benefits to people include: pleasure from contact with nature; feelings of usefulness by providing food; gaining the trust of animals; education of both adults and children; entertainment; aesthetic benefits; and to observe or photograph animals [12,16,27,28,29,30]. 

Ethical reasons can also motivate people, as some people feel that feeding wildlife is a way of counteracting human actions, such as habitat destruction [15], and compensating for a lack of natural foods in urban/suburban environments [11]. The perception that feeding benefits and assists wild animals motivates others [27]. A blurred distinction between wild and domestic animals may be another underlying motive for feeding wildlife [29]. In an unusual case of persistent bear feeding in British Columbia, the feeder expressed a feeling of protection and attachment to “*his*” bears [31], and similar feelings were reported by individuals’ feeding birds in Australia [16]. 

### 2.2. Types of Wildlife Feeding and Outcomes

Four broad categories can be used to describe the types of intentional feeding of free-living wildlife: (1) research, (2) management, (3) tourism and (4) opportunistic.

#### 2.2.1. Research Feeding

The feeding or ‘provisioning’ of free-living wildlife is used occasionally in scientific studies. Natural or novel foods are provided directly (by hand) or indirectly (at feeding stations), sometimes in an attempt to tame or habituate the animals, so that they can be observed and studied more closely. In Gombe, Tanzania, for example, food items were used to habituate chimpanzees to close human proximity in order to facilitate behavioural observations [32]. However, the feeding led to questions about the validity of the observations [33] and to interspecies aggression between chimpanzees and baboons [34], which caused poor welfare in some individuals. 

Supplemental feeding may also be used to answer ecological and biological questions about a species, such as home range size, survival, growth rates, behaviour, reproduction and distribution [25,35,36], by removing or mitigating the effects of food as a limiting factor [24,37]. Feeding experiments with songbirds, for example, have revealed changes in singing and territorial behaviour [38], while provisioned woodland birds were observed to advance their nest construction [39]. Studies involving feeding today are generally short-term, involve small sample sizes, aim to avoid permanent food-conditioning and are overseen by research ethics committees when conducted by academic institutions. Research feeding studies are also important to improve the understanding of other types of intentional feeding to determine if there is broader applicability to other wild populations [17,40].

#### 2.2.2. Management Feeding

Management or ‘supplemental’ feeding can be used to achieve conservation objectives, such as increased survival or reduced human-wildlife conflict. Such prescribed feeding can help to recover or re-establish species [41]. For example, feeding has been used to support the recovery of endangered species, like the Mauritius kestrel [42], bearded vulture [43] and Iberian lynx [44]. However, such efforts are not always without risk, as they can promote disease and infection [45,46]. Feeding is also used as a strategy to reduce human-wildlife conflict [47,48]. Some nature reserves allow for official feeding by reserve staff in an attempt to prevent conflicts that can arise from interactions with tourists who attempt to see (and sometimes feed) the animals; however, such interactions may not necessarily be curtailed by these efforts [49]. 

In North America, large-scale winter feeding of ungulates commonly occurs [50,51] with the intention of preventing deaths, controlling wildlife damage to agriculture and promoting hunting opportunities [52]. The supplemental foods, however, are also available to other species, such as raccoons and skunks. These mesopredators may be attracted to the area and thrive, potentially damaging the ecosystem [53]. In addition, the strategy can directly conflict with government wildlife health recommendations and can unnaturally inflate ungulate populations [54,55]. Further, the practice of baiting (supplemental feeding done to aggregate wildlife for capture or hunting) raises concerns for individual animal welfare and population health [52] and is seen as contradictory to the ethical hunting principle of ‘fair chase’ [56]. The transmission of parasites and disease, such as bovine tuberculosis and chronic wasting disease, at both types of highly frequented feeding sites may in fact be negating conservation goals [57,58,59,60].

#### 2.2.3. Tourism Feeding

Wildlife tourism is a growing industry that provides visitors non-consumptive interactions with wild animals. Feeding can support tourism by making the animals predictably and reliably viewable. Examples of species fed in tourism include: primates that are fed, so that time-constrained tourists in Japan can see a “monkey-on-demand” [61]; brown bears that are led to Finnish-Russian feeding sites to entertain 4,000 visitors annually [62]; komodo dragons whose feeding attracts more than 30,000 visitors to Indonesia annually [63]; salt-water crocodiles lured by staged feeding cruises in Australia [64]; African wildlife drawn to safari lodges by carcass feeding or watering holes [65]; and dolphins, fish, stingrays and sharks that are fed in warmer climates, like Hawaii, Australia, South Africa, Mexico and the Caribbean [66,67,68,69,70]. 

The behavioural and ecological consequences of tourism feeding have been studied among aquatic and land-based species in both protected and non-protected areas. Feeding wild animals can affect both individuals and populations, as animals may experience food-based aggression and social stress [28]. Studies have also documented population-level changes in abundance [66], behaviour and distribution [67], as well as behavioural changes in inter-connected species [68] and overall ecosystem concerns [71].

For individual animals, research has shown that food-conditioned wildlife may suffer nutritionally, become dependent on unreliable food sources [5,72], habituate to people and become more susceptible to predators and vehicle collisions [72,73]. Even when such feeding programs are highly regulated, food-conditioned animals have a high potential for being harmed [74]. Other reported concerns include higher parasite loads [6] and decreased overall health, reproduction and fitness [69]. For example, research into dolphin feeding programs in Australia found increased survival of offspring when anthropogenic feeding decreased [70]. Intentional feeding is particularly concerning in such a highly social species as dolphins, since harmful behaviours can be learned from conspecifics [75].

Proponents claim that tourism feeding is a useful conservation tool to monitor populations [76] and that it promotes indirect conservation benefits through awareness [77]. Others suggest that the effects on individual animals are minimal and that economic benefits to local people are considerable [62,78]. Economic alternatives to tourism, such as hunting or land clearing for agriculture, may in fact be more detrimental to the wildlife, and thus, the net balance of positive and negative effects of wildlife tourism are often hard to determine [77]. In species, such as sharks, the indirect-use value from tourism even helps to ensure that a live animal is more desirable to the local economy than a dead one, benefitting both conservation and welfare [79]. Yet, the long-term animal welfare implications of wildlife feeding should still be studied and included in evaluating the acceptability of feeding. 

In protected areas, feeding may contribute to good local public relations, but it can also devalue nearby wildlife research if animals are unnaturally drawn to people [68]. Even in non-protected areas, wild animals within viewing range are also within a range where they may cause nuisance to local residents [65]. Caution should be taken especially in tourism programs when feeding animals that pose lethal risks to people, as continuous and long-term feeding activities can lead to intra- and inter-species aggression [79]. Feeding can also act as a facilitator to the illegal pet trade, as wild animals habituated to tourists may be more vulnerable to poaching [80].

#### 2.2.4. Opportunistic Feeding

Opportunistic feeding at roadsides, public spaces and in backyards allows individuals to interact closely with wildlife. Feeding wildlife in public locations is generally discouraged, and occasionally prohibited, but often involves species that are perceived as relatively harmless [2,81]. A common scene in North American parks is that of jays or chipmunks begging picnickers for an opportunistic meal [2], whereas in Asia and Africa, primates may be the local beggars [3,23]. The consequences of opportunistic feeding on migration patterns, non-target species, disease transmission and trophic cascades needs more study [17,25,82]. For highly food-conditioned and habituated animals, withdrawal of feeding can lead to increased stress and aggression as food becomes less and less available [2]. 

Wild bird feeding is the most popular form of wildlife interaction in Western culture [83], and its biological and conservation merits continue to be debated [25,38,40]. Provisioning inappropriate foods, the spread of disease at feeders and window strikes are the greatest animal welfare concerns of backyard bird feeding [11,84,85]. Feeding waterfowl is also an international phenomenon, but there is little known about its effects [83] aside from its contribution to environmental degradation and water pollution [71].

Opportunistic backyard feeding of raccoons, squirrels, skunks, bears, coyote and deer (either directly or indirectly via bird feeders) also contributes to poor welfare (as discussed above) and neighborhood nuisance issues in both urban and rural settings. Even before public safety becomes an issue: the animals may be trapped and relocated or killed by concerned residents. The extent of this feeding is difficult to assess, because unlike bird feeding, which can be estimated from the purchases of related supplies, most foods types are also used for human or domestic animal consumption.

In summary, there is little evidence of any benefit to the animals’ long-term welfare from opportunistic feeding. Recent evidence suggests that even winter bird feeding, although widely practised, may even be detrimental to some populations [86], contrary to popular beliefs. Feeding can cause some wildlife to lose their fear of people and associated flight response, leading to nuisance and/or aggressive behaviours [72]. In North America, sensational stories of humans or pets interacting closely with habituated bears and deer are not uncommon and spur considerable public debate over culling [87,88,89]. In a prominent case in Australia, the cull of food-habituated dingoes was ordered by the government after a deadly attack on a child [90].

## 3. British Columbia, Canada—A Case Study of Feeding Wildlife to Death

British Columbia (BC), Canada, has abundant populations of grizzly bear, black bear, moose, elk, deer and coyote, together with organized wildlife viewing opportunities for various species, including whales and eagles. Feeding for research or management is limited [52] and tourism feeding is not officially condoned for any species. Feeding of all “dangerous wildlife” (*i.e.*, bears, cougars and wolves) is prohibited and subject to high fines [91]. Enforcement of this provincial law is complaint-based and currently does not include ungulate species. Backyard bird feeding is a popular pastime regulated only by local municipal bylaws that may seasonally restrict or prohibit feeders, due to the risk of attracting wildlife deemed “dangerous”*.* Generally, feeding of all wildlife in regional, provincial and federal parks is either prohibited or discouraged.

Nonetheless, recent incidents of feeding led to numerous wildlife deaths in the province. In summer, 2011, 24 black bears were killed by officials in the small town of Christina Lake after a high-profile and decade-long case of illegal bear feeding at a private residence [31]. Residents of the community knew about the feeding for years, but failed to see it as a serious form of animal harm, even after an earlier incident, when many bears had been killed [31]. A survey of the community highlighted a lack of education and enforcement on the issue [31]. Between December, 2011, and March, 2013, three communities (Cranbrook, Kimberley and Invermere) conducted controversial urban deer culls, removing 172 mule and white-tailed deer, in an attempt to reduce deer-human conflict [92,93,94,95]. Opportunistic deer feeding by locals is cited as one factor contributing to growing urban ungulate conflict in the province [96]. Unlike some deer culls in the US, however, these culls were not conducted to address risks associated with chronic wasting disease or lime disease, which are non-existent and rare (respectively) in the province [97,98]. Previous deer culls in BC had been limited to islands with sensitive and endangered habitats and sparse human populations [99,100]. An educational program to prevent human-wildlife conflict in BC had previously focused on human-bear interactions. However, due to increased (real or perceived) conflicts, the program has broadened to include other species, rebranded as WildSafeBC [101]. 

To describe the regulatory environment in the province, the authors reviewed wildlife feeding bylaws in BC’s 155 municipalities and found that 72% have no bylaws prohibiting intentional wildlife feeding or managing attractants, like garbage. Bylaws to manage garbage, generally requiring households to use wildlife-resistant containers and/or to put out garbage only on the day of collection, were present in 9% of communities. A variety of feeding bylaws exist in 12% of municipalities; some restrict feeding by species (deer, birds, pigeons and fur-bearers); several ban all feeding in parks; and a few prohibit backyard bird feeding annually between April and October. The final 7% had combined garbage/attractant and feeding bylaws. In summary, the regulation of wildlife feeding with bylaws is low and inconsistent in BC; although a few communities focus on selected problem species, in general, feeding is not seen as an enforcement priority, with few fines levied. 

Overall, BC has fairly restrictive policies on research, management and tourism feeding, but current education and regulations to prevent opportunistic feeding appear minimal. Signage and threats of fines may not be as effective as peer pressure from members of the public who express disapproval of the activity and may be the most promising way to discourage feeding [102]. Opportunistic feeders are often well-intentioned, believing that feeding benefits or causes no harm to animals. However, without negative social feedback, they may not be aware that much feeding is inappropriate. Better management and education campaigns incorporating animal welfare into a framework to evaluate feeding activities may help people to recognize (and hence help to prevent) the harm that feeding often causes. 

## 4. Framework for Evaluating Feeding

We propose that different types of wildlife feeding activities can be evaluated using three factors: the ability to control the activity (C) and its effects on conservation (E) and on the long-term welfare of animals (W) (Table 1). First, the ability to control the activity (regulate, monitor or intervene) is important to ensure that intended outcomes are achieved and to reduce personal safety risks to the public. The positive effects on conservation include contributing to the understanding of the species, saving endangered species and improving population survival. Activities that are of educational or economic value to the local people can also have positive effects on conservation, for example, by giving animals indirect-use economic value. The negative effects on conservation would include facilitating poaching and promoting the spread of disease. The long-term effects on animal welfare are influenced by the number of animals affected, the potential for physiological and physical stress, the duration of feeding relative to an animal’s life expectancy and whether it disrupts natural foraging. We recognize there are differences between individual animals, as well as differences between species, based on their potential to be habituated and to pose a physical threat to humans. Furthermore, research to date has studied the effect of feeding terrestrial species more than aquatic species; however, the framework is intended as a general guideline for assessment, which can be adapted to the many different species and circumstances involved.

**Table 1:** Wildlife feeding acceptability framework: four types of feeding activities evaluated by their ability to be controlled (C) and their effects on conservation (E) and animal welfare (W).

Factors (C, E, W)	Research	Management	Tourism	Opportunistic
C: feasible to regulate/monitor/intervene	+ +	+	−	− −
C: safe for the public	+ +	+	− *	− *
E: contributes to understanding the species	+ +	+	+	−
E: contributes to saving endangered species	+	+ +	−	−
E: contributes to population survival	+ *	+ *	−	−
E: does not facilitate poaching or disease	+	− *	−	− *
E: contributes to public education	N/A	N/A	+/− **	+ *
E: provides economic benefits	N/A	+	+/− **	−
W: effects relatively few animals	+ +	+	− **	− −
W: does not cause physiological stress to animal	+	+	− *	− *
W: does not cause physical harm to animal	+	− *	− *	− *
W: affects only a small portion of lifespan	+ +	+	− − *	− − *
W: does not disrupt natural foraging	+	−	− − *	− − *

Items are rated high (+ +), somewhat high (+), somewhat low (**−**) or low (**− −**), not applicable (N/A) based on general knowledge of the literature. The use of * indicates that the evaluation may vary for different cases; specifically, * = depends on the species involved and ** = depends on the tourism operator.

Using the framework in Table 1, we evaluated several reported examples of each type of feeding (research, management, tourism and opportunistic) in Table 2. We rated the acceptability of each example based on the three factors. For the most part, we deemed a feeding activity acceptable only if it could be controlled, if it had a beneficial conservation effect and if it did not compromise an animal’s long-term welfare. Considerations for the feeding effects on the conservation or welfare of non-target animals were also considered; that is, feeding may be deemed unacceptable if it has negative consequences for other species. 

**Table 2:** Application of the wildlife feeding acceptability framework to reported examples of wildlife feeding based on their ability to be controlled, have beneficial effects on conservation and have a positive long-term effect on animal welfare.

Feeding activity example	Ability to be controlled	Beneficial conservation effect	Positive long-term effect on animal welfare	Feeding acceptable?
*Research*				
Northern Goshawk study [36]	+ +	+	+	Yes
Townsend’s Chipmunk study [37]	+ +	+	+	Yes
Woodland bird study [38]	+ +	+	+	Yes
*Management*				
Kestrel species recovery [42]	+ +	+ +	+ +	Yes
Winter deer feeding [57,58,59]	−	−	−	No
Boar baiting [60]	−	− −	− −	No
*Tourism*				
Dolphin feeding [74]	−	−	−	No
Primate feeding [61]	−	−	−	No
Bear feeding [62]	−	−	−	No
Komodo dragon feeding [63]	−	−	−	No
Shark feeding [79]	+ / −*	+	−	Yes *
*Opportunistic*				
Backyard bear feeding [31]	− −	− −	−	No
Backyard bird feeding [17]	−	+	Neutral	Yes **
Dingo feeding [90]	−	− −	−	No

Items are rated high (+ +), somewhat high (+), somewhat low (**−**), low (**− −**) or neutral; * depends on tourism operator; ** acceptable with conditions: appropriate food by species and season, prevention of non-target species attraction, does not increase the risk of predation (e.g., from cats) or of window strike and does not increase intra- or inter-species aggression.

## 5. Discussion

According to the criteria proposed, many research and management feeding programs would appear acceptable, because they can be controlled, are intended to benefit populations and may improve individual welfare. In contrast, most baiting intended to increase hunting opportunities would be judged unacceptable, because it does not benefit the animals’ long-term welfare or conservation, is difficult to control and may expose both target and non-target animals to disease and increased human-wildlife conflict [41,53]. There is a need for wildlife managers to clearly communicate the objectives and benefits of feeding programs so as to distinguish acceptable feeding, notably in research and management, from other types. 

In most of the tourism examples evaluated, feeding was deemed unacceptable. Even in highly regulated activities with relatively harmless animals, any short-term benefit to the animals’ welfare appeared to be far outweighed by the negative long-term effects of poor physical and psychological health and the production of unnatural behaviours. Understandably, feeding in tourism is appealing to both tourists and companies, because it can increase the potential of seeing otherwise elusive and exotic animals [72]. However, encouraging the feeding of certain animals in certain places, as in tourism feeding, can contribute to public misunderstanding about the overall risks of wildlife feeding, e.g., [23].

Opportunistic feeding often leads to negative welfare and/or human-wildlife conflicts for mammals and some bird species, in addition to being poorly controlled and serving no conservation purpose. As seen in the BC case studies, the feeding of deer and bears (as well as many other species) will continue to trouble communities without effective local bylaws, enforcement and education. Such feeding can lead to animals being culled or relocated, with negative effects on their welfare [103]. These traditional conservation tools, targeting wildlife rather than human behaviour, have limited short-term success and may not be accepted by the public [104]. There is an ongoing need for research to measure the effectiveness of communication, education and links between attitudes and behaviour-modification to improve programming over time [105,106]. Repeat feeding offenders need to be monitored and fined consistently and community support against feeding encouraged, as, often, locals are aware of the problem before the authorities are [31].

Wildlife feeding is often claimed to be an enjoyable and beneficial conservation activity. According to the analysis proposed above, feeding is unacceptable in a great many circumstances. The variety of possible feeding interactions, the range of underlying motivations, the benefits and risks to animals and the inconsistency of approaches to restrict feeding present a confusing situation for wildlife managers. The framework presented here could help managers and educators communicate with the public about which types of feeding are acceptable and unacceptable. This would improve the current status of mixed messages regarding feeding; for example, the acceptability of winter supplemental feeding and feeding exotic animals as a tourist attraction, when similar feeding in parks or backyards is discouraged.

Currently, options for managing wildlife feeding include prohibition, ignoring the problem or managing the feeding [72]. However, bans are unrealistic for some types of feeding, and current approaches towards regulated and unregulated feeding programs lack consistency. A more promising approach may be to change public perceptions about wildlife feeding through repeated education and regular enforcement. Forms of feeding that are dangerous to animals, for example, by creating disease risk or human-wildlife conflict, need to become socially unacceptable. The proposed evaluative framework may assist policy-makers, educators and wildlife managers in establishing which feeding is acceptable, so that unacceptable forms can be targeted through regulations and social pressure.

## 6. Conclusions

In summary, many wildlife feeding activities lead to problems of public safety, conservation and animal welfare. By considering these types of effects in combination, managers and policy-makers may be able to identify acceptable and unacceptable forms of wildlife feeding as a basis for regulations, public education and enforcement.
